# Food Waste-Derived Activated Carbon-Modified Nb_2_CT_x_ MXene for Supercapacitor Applications

**DOI:** 10.3390/nano16060349

**Published:** 2026-03-12

**Authors:** Mehmet Topuz, Fatma Coskun Topuz

**Affiliations:** 1 Department of Mechanical Engineering, Van Yuzuncu Yil University, Van 65080, Turkey; 2 Department of Nutrition and Dietetics, Hakkari University, Hakkari 30000, Turkey; fatmacoskun@hakkari.edu.tr

**Keywords:** food waste, activated carbon, Nb_2_CT_x_ MXene, supercapacitor

## Abstract

In this study, the structural and electrochemical performance of Nb_2_CT_x_ MXene-based composite electrodes modified with activated carbon (AC) derived from food waste was systematically investigated for supercapacitor applications. Three composites with Nb_2_CT_x_:AC mass ratios of 90:10 (MXAC1), 80:20 (MXAC2), and 70:30 (MXAC3) were prepared and comparatively evaluated. SEM/EDS, XRD, HR-TEM, XPS, and BET analyses revealed that, in the MXAC2 composite, activated carbon was homogeneously distributed between the MXene layers, effectively suppressing restacking and promoting the formation of a hierarchical micro/mesoporous structure. XPS results confirmed the preservation of the Nb–C framework and the enrichment of surface functional groups (–O, –OH, and –F). BET analysis demonstrated that MXAC2 possesses an optimized pore architecture that facilitates efficient ion diffusion. Electrochemical measurements revealed that the MXAC2 electrode exhibited the highest specific capacitance at all scan rates and current densities. At 5 mV·s^−1^, MXAC2 achieved a specific capacitance of 651.84 F·g^−1^ and maintained a substantial capacitance even at a high current density of 4 A·g^−1^. EIS analysis confirmed the very low charge transfer resistance (0.023 Ω) and enhanced capacitive behavior for MXAC2. Additionally, MXAC2 has high cycle stability, demonstrating 82.15% capacitive retention and 92.45% coulombic efficiency after 10000 cycles. These results indicate that food waste-derived AC-optimized Nb_2_CT_x_ MXene composite materials are a strong candidate for sustainable and high-performance supercapacitor electrodes.

## 1. Introduction

Supercapacitors have emerged as a promising candidate for next-generation energy storage devices because of their unique capabilities between traditional capacitors and rechargeable lithium-ion batteries. Their high power density, fast charge–discharge speed, and stability in multi-cycle applications make them a preferred choice, particularly for specific applications such as flexible/wearable electronics [1,2,3]. Despite these advantages, the energy density of supercapacitors still lags behind that of lithium-ion batteries. Therefore, recent research has focused on developing next-generation electrodes that can provide high energy density without compromising high cycle stability and fast charge–discharge capabilities. Two main strategies are followed to increase the energy density of supercapacitors. The first is defined as the development of pseudo-capacitive materials to enable faradaic charge storage. The second is defined as the creation of porous electrodes with a high surface area that facilitate electrical double-layer capacitance (EDLC) by enabling efficient ion exchange between the electrode and the electrolyte. Accordingly, current research efforts aim to integrate these two mechanisms by developing electrode materials that simultaneously exhibit strong pseudocapacitive behavior and enhanced EDLC performance.

First reported in 2011, two-dimensional (2D) MXene materials have attracted significant interest in electrochemical energy storage and catalysis due to their outstanding pseudocapacitive properties. MXenes are typically synthesized through the selective chemical etching of the layer from MAX phases [2]. Depending on their composition, MXenes can be classified as carbides, carbonitrides, or nitrides. Owing to their surface terminations (T_x_ groups, such as –O, –OH, and –F), 2D MXenes exhibit high electrical conductivity, tunable surface chemistry, and efficient ion adsorption–desorption behavior [2,4]. These surface functionalities make MXenes highly suitable for the fabrication of high-performance supercapacitor electrodes [2,5].

Studies have been conducted on various MXene compositions derived initially from the Ti_3_AlC_2_ MAX phase. Among the wide range of MXene variants, niobium-based MXenes (Nb_2_CT_x_) have attracted increasing attention in supercapacitor applications because of their enhanced redox activity and high intrinsic electrical conductivity. These characteristics enable faster ion adsorption–desorption kinetics and stronger pseudocapacitive behavior compared to Ti-based MXenes. Furthermore, reversible Nb oxidation state transitions contribute to additional Faradaic charge storage, thereby improving the overall capacitance. With advancements in selective etching strategies, including LiF–HCl treatment, molten salt-assisted synthesis, and electrochemical etching, the structural quality, scalability, and electrochemical performance of Nb_2_CT_x_ MXenes have been significantly enhanced [6,7].

Despite the aforementioned advantages, MXene materials suffer from a critical limitation: restacking. This phenomenon originates from strong van der Waals interactions between adjacent MXene layers, which reduce the interlayer spacing. As a result, the accessible surface area for ion transport decreases, thereby limiting both pseudocapacitive and EDLC contributions and hindering rapid charge transfer [8]. To address this issue, researchers have focused on intercalating porous nanostructured materials with high surface areas and strong ion adsorption capabilities. Recent advances demonstrate that biomass-derived carbons play a crucial role in next-generation electrochemical energy storage systems due to their tunable porosity, heteroatom functionality, and sustainability advantages. In particular, recent reports in [9,10] highlight the strategic integration of hierarchical biomass carbons in high-performance hybrid capacitive systems, further emphasizing the importance of rational pore engineering and surface chemistry optimization. Activated carbons (ACs), known for their high specific surface area, well-developed porosity, mechanical robustness, and chemical stability, are particularly suitable for interlayer modification of MXene structures [11]. The incorporation of AC between MXene layers not only physically prevents restacking but also enhances electrolyte ion diffusion and improves EDLC performance. Consequently, MXene–AC hybrid electrodes exhibit enhanced electrical conductivity, improved pseudocapacitive behavior, and superior cycling stability compared to pristine MXene electrodes [11,12,13].

Activated carbon (AC) is preferred not only as a physical spacer between MXene layers but also for energy storage applications because of its cost-effectiveness, renewability, and environmentally friendly nature. Various biomass-derived precursors, including coconut shells, walnut shells, agricultural residues, fruit peels, and other food wastes, can be converted into AC through controlled carbonization and activation processes [14,15]. In this study, banana peel waste was selected as the biomass precursor owing to its high lignocellulosic carbon content, inherent mineral-assisted activation behavior, and wide availability as an abundant agricultural by-product, making it a promising sustainable carbon source. The presence of oxygen-containing functional groups and hierarchical pore structures enhances electrolyte accessibility and facilitates efficient ion transport within MXene-based electrodes. Consequently, AC derived from food waste offers a sustainable strategy that simultaneously improves electrochemical performance and reduces the environmental footprint of energy storage materials. Moreover, such biomass-derived carbons contribute to MXene-based composites in two primary ways: (i) by promoting synergistic ion adsorption and interfacial interaction with MXene layers and (ii) by offering a low-cost, environmentally sustainable alternative to conventional carbon sources.

In scientific research, carbon nanotubes, graphene, carbon black, and activated carbons (ACs) are widely employed as carbon-based materials to achieve hybridization and enhance the synergistic interactions within MXene-based systems. These hybrid architectures effectively mitigate the restacking tendency of MXene layers and exhibit improved electrochemical performance due to enhanced ion diffusion kinetics [16]. Furthermore, Nb_2_CT_x_-based hybrid materials incorporating transition metal oxides, hydroxides, or conductive carbons demonstrate strong pseudocapacitive behavior, enhanced redox activity, and improved ion transport pathways [17]. These findings highlight the critical role of interface engineering, surface functionalization, and pore architecture in the rational design of next-generation MXene-based hybrid electrodes.

Addressing these challenges is essential for translating MXene-based hybrid materials into practical and scalable supercapacitor technologies. For example, De et al. [18] investigated polyindole-stabilized, nanocellulose-wrapped Ti_3_C_2_T_x_ MXene nanocomposites to suppress restacking and enhance electrical conductivity. The optimized composition achieved a specific capacitance of 858 F·g^−1^, while the assembled asymmetric device delivered 90 F·g^−1^, an energy density of 40.5 Wh·kg^−1^, and 94.1% capacitance retention over 10,000 cycles, demonstrating a low-cost bio-waste-derived electrode strategy. Similarly, Zhou et al. [19] prepared nitrogen-doped activated carbon derived from sugarcane tips and incorporated it into MXene/AC composite membranes to mitigate stacking, enhance ion transport, and increase the number of electrochemically active sites. In addition, Yu et al. [20] reported a one-step fabrication strategy for flexible MXene-bonded AC electrodes, in which Ti_3_C_2_T_x_ MXene simultaneously functioned as a binder, conductive backbone, and active material. The encapsulated carbon structure and expanded MXene interlayer spacing formed a three-dimensional conductive network, enabling high capacitance (126 F·g^−1^) and strong rate capability for flexible supercapacitor applications. Furthermore, Sinha et al. [21] utilized orange peel-derived activated carbon as a sustainable and low-cost spacer to prevent MXene restacking. The resulting composite exhibited expanded interlayer spacing and improved electrochemical performance (407 F·g^−1^), while the all-solid-state device retained 92.98% of its capacitance after 5000 cycles. Despite these advances, there remains a need for systematic investigation of hybrid structures based on different material combinations. In particular, the optimal mass ratio of food-waste-derived activated carbon in Nb_2_CT_x_ MXene composites has not been comprehensively explored, which constitutes the primary motivation for this study. Moreover, while previous studies have predominantly focused on microscale porous AC structures, nanoscale AC–MXene hybrid architectures remain insufficiently examined, further emphasizing the significance of the present work [22,23]. Recent studies published in [24,25] have demonstrated that rational interface engineering and hierarchical hybridization strategies significantly enhance ion transport kinetics and structural stability in advanced supercapacitor systems. These findings further support the importance of optimizing MXene–carbon interactions, as demonstrated in the present study.

In this work, a systematic approach is presented for developing a high-performance Nb_2_CT_x_ MXene composite modified with food waste-derived AC. The study involves the optimized synthesis of Nb_2_CT_x_, the controlled preparation of AC from an abundant agricultural food waste precursor, and the fabrication of MXene–AC composites designed to enhance interlayer spacing, structural integrity, and electrolyte accessibility. Extensive characterization, including SEM/EDS, XRD, HR-TEM, XPS, and BET analyses, was conducted to confirm the structural and physicochemical properties of the materials and correlate them with electrochemical performance. Electrochemical performance was evaluated using cyclic voltammetry (CV), galvanostatic charge–discharge (GCD), and electrochemical impedance spectroscopy (EIS) to investigate pseudocapacitive behavior, double-layer contributions, ion-transport resistance, and long-term cycling stability. The combined results demonstrate a sustainable, low-cost, and high-efficiency electrode architecture capable of advancing scalable supercapacitor technologies by integrating the pseudocapacitive properties of MXene with the hierarchical porosity and eco-friendly characteristics of food waste-derived AC.

## 2. Materials and Methods

### 2.1. Materials

Niobium aluminum carbide (Nb_2_AlC, >99%, 325 mesh, Nanografi, Ankara, Turkey) MAX phase, hydrofluoric acid (HF, 37 wt.% aqueous solution, Sigma–Aldrich, St. Louis, MO, USA, CAS: 7664-39-3), potassium hydroxide (KOH, ≥85%, pellets, Merck, Darmstadt, Germany, CAS: 1310-58-3), N-methyl-2-pyrrolidone (NMP, ≥99.5%, anhydrous, Sigma–Aldrich, CAS: 872-50-4), poly(vinylidene fluoride) (PVDF, powder, Sigma–Aldrich, CAS: 24937-79-9), potassium chloride (KCl, ≥99%, Merck, CAS: 7447-40-7), and all other reagents were purchased from commercial suppliers and used without further purification. Fresh banana peels were collected from local sources, thoroughly rinsed with deionized water (DW), and used as the food waste precursor for AC.

### 2.2. Preparation of Biochar and Activated Carbon

**Figure 1:**
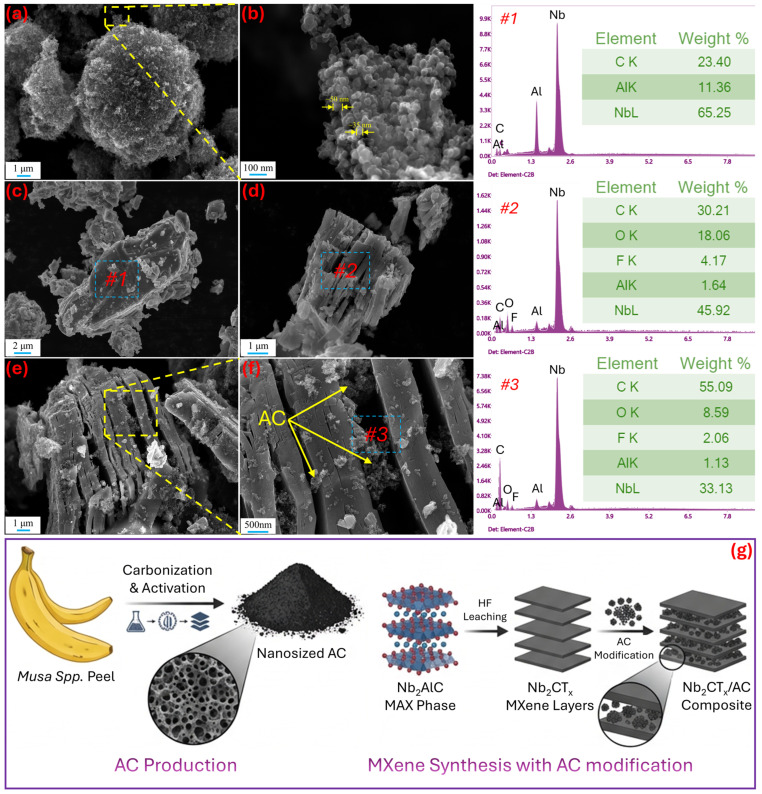
SEM images: (**a**,**b**) AC, (**c**) Nb_2_AlC MAX phase, (**d**) Nb_2_CT_x_ MXene, and (**e**,**f**) MXAC2 composite electrode; regions marked *#1*, *#2*, and *#3* show the EDS spectra of MAX, MXene, and MXAC2 samples, respectively. (**g**) Production of AC and modification steps of MXAC composites.

Banana peels were thoroughly washed with deionized water (DW) to remove adhering dirt and soluble sugars, then cut into approximately 2 cm pieces and oven-dried at 80 °C for 24 h until constant weight was achieved. Dried banana peel pieces (10 g per batch) were placed in a tubular furnace under a continuous N_2_ flow (200 mL·min^−1^). The furnace temperature was increased to 500 °C at a heating rate of 5 °C·min^−1^, maintained for 2 h, and subsequently cooled to room temperature under an N_2_ atmosphere. The obtained material (biochar) was mixed with KOH at a mass ratio of 1:2 (biochar:KOH). The mixture was homogenized by grinding in an agate mortar and impregnated with a minimal amount of DW to form a uniform paste. The paste was dried at 110 °C overnight. The dried mixture was then heated in a tubular furnace under N_2_ to 700 °C at a rate of 5 °C·min^−1^ and held for 1 h. The present study focuses on composite optimization at a controlled activation temperature (700 °C), while a systematic investigation of temperature-dependent activation (700–900 °C) and its effect on MXene dispersion will be addressed in future work [22]. After cooling under N_2_, the activated product was washed repeatedly with 0.1 M HCl to remove residual potassium species and subsequently rinsed with DW until a neutral pH (~7) was reached. The washed sample was dried at 110 °C for 12 h and ground into a fine powder, denoted as AC (banana peel-derived activated carbon). The AC powder was further subjected to particle size reduction using a planetary ball mill equipped with tungsten carbide balls at a ball-to-powder ratio of 15:1. Milling was performed at 450 rpm for 18 h under an Ar atmosphere, using 10 min milling and 10 min rest cycles to prevent overheating. A small amount of ethanol was added as a dispersant to minimize particle agglomeration. The resulting nano-sized AC powder was dried at 80 °C, yielding particles in the approximate size range of 10–100 nm (Figure 1b). Table 1 summarizes the CHNS elemental composition (Thermo Fisher Scientific, FLASH 2000, Waltham, MA, USA) of the banana peel-derived AC, providing quantitative information on carbon, hydrogen, nitrogen, and sulfur contents. The elemental composition is consistent with previously reported studies, where chemical activation of banana peel biomass led to increased carbon content and reduced heteroatom concentrations [26,27].

### 2.3. Synthesis of Nb_2_CT_x_ MXene

Nb_2_CT_x_ MXene was synthesized via selective etching of the Al layer from the Nb_2_AlC MAX phase. Briefly, 1.0 g of Nb_2_AlC powder was slowly added to 20 mL of 48 wt.% hydrofluoric acid (HF) in a PTFE beaker under continuous magnetic stirring. To selectively remove the Al layers and obtain multilayer Nb_2_CT_x_, the reaction was conducted at 35 °C for 24 h. After etching, the reaction mixture was repeatedly washed by centrifugation at 3500 rpm for 5 min per cycle using deionized water until the supernatant reached a pH of approximately 6. The resulting multilayer Nb_2_CT_x_ sediment was collected and dried at 70 °C for 24 h.

### 2.4. Preparation of Active Material

Composites were prepared by physically mixing multilayer Nb_2_CT_x_ suspension with AC powder. The active material (AM) mass ratios of Nb_2_CT_x_ to AC were adjusted to 90:10 (MXAC1), 80:20 (MXAC2), and 70:30 (MXAC3) to optimize the balance between electrical conductivity and porosity. For the 80:20 composite (MXAC2) as a representative example, the required amount of AC was first dispersed in an ethanol/DW mixture (70:30 vol.%) by sonication for 10 min. The dispersion was then added to an aqueous Nb_2_CT_x_ colloidal suspension under gentle stirring and mixed for 2 h to ensure homogeneous interaction. The resulting suspension was collected by centrifugation at 5000 rpm for 10 min. The precipitate was washed three times with ethanol to remove residual impurities and loosely bound particles. Finally, the collected material was dried in a vacuum oven at 60 °C for 12 h, yielding the composite active material powder for subsequent characterization and electrochemical measurements.

### 2.5. Electrode Preparation

Working electrodes were prepared by drop-casting a slurry of the active material (AM) onto a glassy carbon electrode (GCE, 3.0 mm in diameter). The electrode ink consisted of 90 wt.% AM and 10 wt.% PVDF binder. PVDF was first dissolved in N-methyl-2-pyrrolidone (NMP) to prepare a 5 wt.% PVDF solution. The AM was then added to the PVDF/NMP solution to obtain an ink concentration of approximately 10 mg·mL^−1^, followed by ultrasonication for 30 min to ensure homogeneous dispersion. The resulting ink was drop-cast onto the polished GCE surface using a micropipette to achieve a mass loading of 1 mg·cm^−2^. After drop-casting, the electrodes were dried and weighed using an analytical balance (±0.1 mg accuracy) to confirm the actual mass loading. The deviation between samples was within ±5%, ensuring acceptable reproducibility.

### 2.6. Characterization

Morphology and microstructure were examined using a field-emission scanning electron microscope (FE-SEM, Zeiss Sigma 300, Jena, Germany). Before imaging, the samples were sputter-coated with a thin Au/Pd conductive layer to prevent surface charging. High-resolution transmission electron microscopy (HR-TEM, JEOL 2100, Peabody, MA, USA) was performed at an accelerating voltage of 200 kV. For HR-TEM analysis, the samples were dispersed in ethanol, drop-cast onto lacey carbon-coated copper grids, and dried under ambient conditions. X-ray diffraction (XRD, PANalytical Empyrean, Malvern, UK) patterns were recorded using Cu-Kα radiation (λ = 1.5406 Å) over a 2θ range of 5–80°, with a step size of 0.02° and a scanning rate of 1°·min^−1^ to identify phase composition and evaluate the basal spacing of Nb_2_CT_x_ structures. Surface chemical states were analyzed by X-ray photoelectron spectroscopy (XPS, Thermo Scientific K-Alpha, Waltham, MA, USA) using an Al Kα radiation source. The binding energies were calibrated regarding the C 1s peak, and the core-level spectra of Nb, O, and surface termination groups (–O, –OH, –F) were deconvoluted to quantify the surface chemical composition. Measurements of N_2_ adsorption–desorption at 77 K, employing the Brunauer–Emmett–Teller (BET) method (Micromeritics TriStar II Plus, Malvern, UK), were used to determine the specific surface area and pore size distribution. Before analysis, the samples were degassed under vacuum at 150 °C for 12 h.

### 2.7. Electrochemical Measurements

Electrochemical characterization was performed in a three-electrode cell at room temperature (25 ± 2 °C) using a PC-controlled potentiostat/galvanostat (Emstat 4R, Palmsens, Houten, The Netherlands). The modified GCE was the working electrode; a platinum wire (Pt) served as the counter electrode, and a silver/silver chloride (Ag/AgCl: saturated KCl) electrode was used as the reference electrode. All potentials are reported vs. Ag/AgCl in 1.0 M KOH (aqueous). A 1 M KOH electrolyte concentration was selected to evaluate intrinsic electrode kinetics under moderate ionic conductivity conditions and to minimize possible MXene surface degradation that may occur at higher electrolyte concentrations, such as 6 M KOH. Cyclic voltammetry (CV) was recorded at scan rates of 5, 15, 30, 40, 50, 100, and 200 mV·s^−1^, over a potential window of −0.2 to 0.5 V vs. Ag/AgCl (window chosen to avoid water electrolysis and match MXene stability). CV was used to probe capacitive and pseudocapacitive behavior. Galvanostatic charge discharge (GCD) tests were performed at 0.25, 0.5, 1, 2, and 4 A·g^−1^ (based on AM mass) current densities, respectively. Long-term cycling was evaluated by repeated GCD at 4 A·g^−1^ for 10,000 cycles, monitoring capacitance retention and coulombic efficiency. Specific capacitances from both CV and GCD curves were calculated with Equations (1) and (2) [19]. Capacitance retention (*CR*, %) and Coulombic efficiency (*CE*, %) were calculated from the GCD curves according to Equation (3) and Equation (4), respectively [19].

(1) $$C_{CV}=\frac{1}{mv\Delta V}\int_{V_{1}}^{V_{2}}I\left(V\right)dV$$

(2) $$C_{GCD}=\frac{I\;\Delta t}{m\Delta V}$$

(3) $$CR\left(\%\right)=\frac{C_{n}}{C_{0}}\times 100$$

(4) $$CE(\%)=\frac{t_{discharge}}{t_{charge}}\times 100$$

m: mass of active material in electrode (g), ν: scan rate (V·s^−1^), $\Delta V=V_{2}-V_{1}$: potential window (V), I(V): current as a function of potential (A), $V_{1},V_{2}$: lower and upper potential limits (V), I: applied discharge current (A), Δt: discharge time (s), $C_{n}$: specific capacitance after the $n^{\mathrm{th}}$ cycle (F·g^−1^), $C_{0}$: initial specific capacitance (F·g^−1^), $t_{discharge}$: discharge time (s), $t_{charge}$: charge time (s). Electrochemical impedance spectroscopy (EIS) spectra were recorded from 100 kHz to 10 mHz with an AC amplitude of 5 mV at OCP. Nyquist plots were used to extract equivalent circuit diagrams and charge-transfer characteristics. All electrochemical measurements were performed at least in triplicate using independently prepared electrodes. Mass loadings, geometric areas, and current densities are explicitly reported to facilitate reproducibility.

## 3. Results and Discussion

Figure 1 presents SEM images of the AC obtained from banana peel waste, Nb_2_AlC MAX, Nb_2_CT_x_ MXene, and MXAC2 composite samples used in the study, along with EDS analysis results from selected *#* regions. The SEM images of nano-sized AC powder taken at higher magnifications in Figure 1b confirm that the micrometer-sized irregular agglomerates are formed by the aggregation of nanometer-scale primary carbon particles (Figure 1a). This morphology, with high roughness and heterogeneity, indicates the advanced porous structure and large specific surface area of the AC [28]. It also suggests the presence of favorable active sites for the adsorption of its ions in a KOH electrolyte. The SEM image of the Nb_2_AlC MAX phase used to obtain the Nb_2_CT_x_ MXene phase is given in Figure 1c. Dense, compact, and layered morphology was observed in the MAX phase. According to the *#1* EDS analysis, the MAX phase revealed the presence of Nb, Al, and C elements in the order of 65.25, 11.36, and 23.40 wt.%, respectively. This elemental composition is consistent with the theoretical stoichiometry of the Nb_2_AlC MAX phase [29]. Therefore, the initial MAX phase is suitable for the chemical leaching process. The SEM image of the Nb_2_CT_x_ MXene structure presented in Figure 1d also supports this phenomenon. After chemical leaching, an accordion-like layered morphology is formed compared to the stacked topsoil’s MAX phase. Therefore, the Nb_2_CT_x_ MXene layers open up, increasing surface area. This phenomenon is significant because of the multiple contact points that will form with the KOH electrolyte [30]. The decrease in Al to 1.64 wt.% in the EDS analysis taken from region *#2* of the Nb_2_CT_x_ MXene indicates the successful completion of the chemical leaching process.

The accordion-like morphology, which is formed by the absence of the Al element, especially in the middle sections during the MAX phase, prevents further exfoliation of MXene and provides structural integrity. The O and F elements were found on the Nb_2_CT_x_ MXene surface at 18.06 wt.% and 4.17 wt.%, respectively. MXene synthesis also stems from HF leaching. This indicates that the MXene surface is terminated with functional groups such as –O/–OH and –F, and that the typical Nb_2_CT_x_ MXene chemistry has been successfully achieved [31]. SEM images of the MXAC2 sample, which exhibits high electrochemical performance, are shown in Figure 1e,f. AC nanoparticles are clearly observed to be homogeneously distributed on the MXene surface and between the layers. Therefore, it is believed that the presence of AC suppresses the disadvantage of MXene’s re-stacking, contributing to the formation of a hierarchical structure with a higher surface area [32]. Similarly, the EDS analysis of the region marked *#3* on MXAC2 shows an increase in C content to 55.09 wt.% compared to the Nb_2_AlC MAX and Nb_2_CT_x_ MXene, which also confirms the presence of AC throughout the structure. Therefore, it indicates that AC was successfully integrated into the MXene structure and could play a role in facilitating electron conduction and ion diffusion by forming a conductive carbon network. It is considered that this morphological and chemical synergy forms the basis of the superior electrochemical performance of the MXAC2 composite electrode. All AC production and Nb_2_CT_x_ MXene synthesis with AC modification steps are visually represented in Figure 1g.

**Figure 2:**
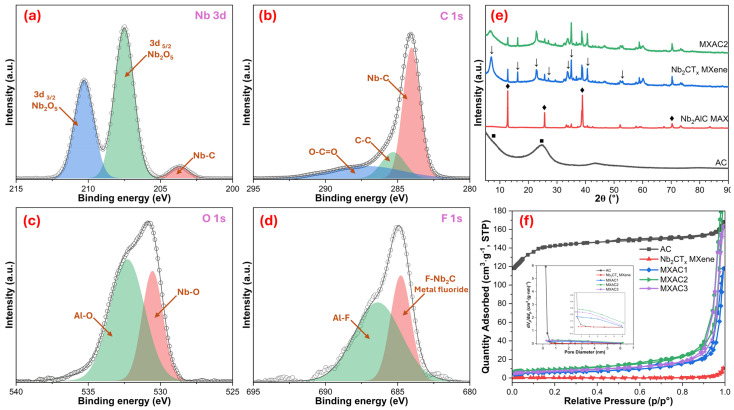
High-resolution XPS spectra of MXAC2 composite: (**a**) Nb 3d, (**b**) C 1s, (**c**) O 1s, and (**d**) F 1s; (**e**) XRD patterns of AC (■), Nb_2_AlC MAX (♦), Nb_2_CT_x_ MXene (↓), and MXAC2 samples; (**f**) N_2_ adsorption–desorption BET isotherm for all electrodes and pore size distribution as an inset.

In Figure 2, the surface chemistry, crystal structure, and porosity properties of the MXAC2 composite are quantitatively presented using XPS, XRD, and BET analyses. In the high-resolution XPS spectrum of Nb 3d shown in Figure 2a, the Nb–C bond observed at 203.62 eV is attributed to the Nb_2_O_5_ component at Nb 3d_5/2_ at 207.47 eV, while the bond at 210.33 eV is attributed to the formation of Nb_2_O_5_ corresponding to 3d_3/2_ [33]. It has been shown that there are partial oxidation and oxygen-based terminations on the MXene surface, especially after contact with the atmosphere [34]. Therefore, it was revealed that the Nb–C skeleton was preserved, but the surface was enriched with electrochemically active functional groups. In the C 1s spectrum presented in Figure 2b, the dominant peak at 284.14 eV is attributed to the Nb–C bond, while the component around 285.38 eV is attributed to the C–C bond. It is emphasized that the observed weak and broad peak at higher binding energy (287.96 eV) represents the O–C=O species [35]. This distribution indicates that activated carbon forms a conductive carbon matrix in the MXAC2 composite, providing a strong interfacial interaction with the MXene. Furthermore, it should also be noted that oxygen-containing functional groups originating from the activated carbon (such as C–O and O–C=O) are mainly reflected in the C 1s spectrum, whereas the O 1s signal in the composite is dominated by Nb–O and Al–O species associated with the MXene structure. The O 1s spectrum in Figure 2c was found to contain components corresponding to the Nb–O bond at 530.56 eV and the Al–O or –OH species at 532.32 eV [36]. These oxygenated functional groups are expected to contribute in a way that can support pseudocapacitive contribution by increasing the surface interaction with the electrolyte. In the F 1s spectrum shown in Figure 2d, the peak at 684.82 eV is attributed to F–Nb_2_C or metal fluoride species, while the broad shoulder peak at 686.40 eV indicates an Al–F bond, confirming the presence of –F terminations on the MXene surface, which are specific to the HF-based etching process [37].

The XRD patterns presented in Figure 2e clearly show the significant differences in the crystal structures of AC, the Nb_2_AlC MAX phase, and the derived Nb_2_CT_x_ MXene, indicating the successful transformation of the MXene. In the XRD pattern of the AC, the broad and low-intensity peaks observed around approximately 2θ at ~25° and ~43° indicate an amorphous-semicrystalline carbon structure [15]. These broad peaks indicate that the AC contains irregular graphitic regions and that the energy storage mechanism can occur predominantly through EDLC. On the other hand, in the XRD pattern of the Nb_2_AlC MAX phase, the sharp and high-intensity peaks observed around 2θ = 13.1°, 26.5°, 39.0°, and 41.6° are associated with the (002), (004), (101), and (104) crystal planes, respectively [38]. These sharp peaks confirm that the MAX phase has a well-crystallized, layered, and ordered structure. It is observed from the XRD pattern of Nb_2_CT_x_ MXene that the high-angle peaks specific to the MAX phase are largely absent, or their intensities are significantly reduced. In contrast, the (002) peak has shifted significantly toward lower angles, appearing at approximately 2θ ≈ 6–7° [39]. According to Bragg’s law (λ = 1.5406 Å), this corresponds to an expanded interlayer spacing (d002) of approximately 1.2–1.4 nm, confirming the successful removal of Al layers and interlayer expansion of Nb_2_CT_x_. This peak shift indicates an increase in interlayer spacing, which is attributed to the removal of Al layers and the entry of surface functional groups (–O, –OH, –F) between the Nb_2_C layers. It is observed from the XRD pattern of Nb_2_CT_x_ MXene that the high-angle peaks specific to the MAX phase are largely absent or significantly reduced, although weak reflections associated with residual Nb_2_AlC may still be detected. With the MXAC2 composite, while the characteristic (002) peak of Nb_2_CT_x_ MXene is preserved, the peak intensity is significantly reduced compared to pure MXene, and the peak is broadened. This situation indicates that the AC is embedded between the MXene layers, suppressing restacking and partially disrupting the interlayer order to create a more open structure [40]. Furthermore, the MXAC2 pattern shows that the broad amorphous band of AC overlaps with the MXene peaks, confirming that the crystalline Nb_2_CT_x_ MXene phase and the AC phase are both present in the composite. The BET results presented in Figure 2f compare the textural properties of pure AC, Nb_2_CT_x_ MXene, and MXAC composites. All samples exhibit type-IV isotherms with hysteresis loops, confirming their mesoporous nature [41]. Pure AC shows a high specific surface area (450.80 m^2^·g^−1^), whereas pristine Nb_2_CT_x_ displays a very low value (0.88 m^2^·g^−1^) due to sheet restacking. Upon composite formation, the surface areas vary systematically—20.95 m^2^·g^−1^ (MXAC1), 33.06 m^2^·g^−1^ (MXAC2), and 26.42 m^2^·g^−1^ (MXAC3)—indicating a composition-dependent optimization effect. MXAC2 exhibits the highest accessible surface area, suggesting that the optimal AC content effectively suppresses MXene restacking while preserving the mesoporous framework. In contrast, lower or higher MXene ratios likely induce partial pore blockage or aggregation, reducing effective porosity. The pore size distribution is centered on ~3–4 nm, facilitating efficient ion transport [42]. The BET pore size distribution of the MXAC2 composite reveals a hierarchical micro/mesoporous structure. Considering that the hydrated diameters of K^+^ (~0.66 nm) and OH^−^ (~0.30–0.36 nm) ions in the KOH electrolyte fall within the sub-nanometer range, these micropores are sufficiently accessible for electrolyte ions. The mesoporous channels further facilitate rapid ion transport and electrolyte diffusion within the electrode structure. This optimized balance between surface area, pore architecture, and interfacial interaction explains the superior electrochemical performance of MXAC2 compared to pure AC and other composites.

**Figure 3:**
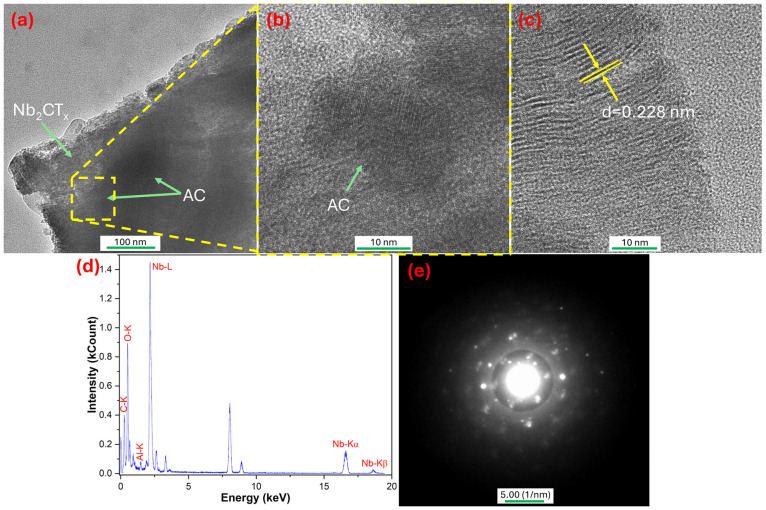
(**a**–**c**) HR-TEM images of MXAC2 powder, (**d**) EDS spectrum, and (**e**) SAED pattern.

The nanoscale structure of the MXAC2 composite, which exhibited high electrochemical performance in Figure 3, was examined using HR-TEM, EDS, and selected area electron diffraction (SAED) analyses. The HR-TEM image obtained at low magnification in Figure 3a clearly shows that AC is distributed at different points across the surface and interior of the Nb_2_CT_x_ MXene. It is observed that the AC phase is concentrated particularly in the edge and surface regions of the MXene layers. It is thought that this situation is entirely because of the incomplete exfoliation of the Nb_2_CT_x_ MXene. Furthermore, it can be shown as indirect evidence that AC suppresses restacking by filling the interlayer spaces and creating a more open structure. In the HR-TEM image taken at higher magnification in Figure 3b, it is observed that the amorphous nature of AC (as seen in the XRD patterns presented in Figure 2e) coexists with the ordered lamellar structure of the MXene layers. The amorphous AC phase provides continuity for electron transport by forming a conductive carbon network between the MXene layers, while also contributing to open ion diffusion pathways [43] and increased surface area (as shown in the BET graph in Figure 2f). In the HR-TEM image presented in Figure 3c, a distinct lattice on the Nb_2_CT_x_ MXene layers is clearly visible. The lattice spacing of 0.228 nm corresponds to the interatomic fringes of Nb_2_CT_x_ MXene as reported earlier [44]. Elemental analysis of the MXAC2 composite is confirmed by the EDS spectrum in Figure 3d, indicating that Nb, C, and O are the most abundant elements. The presence of characteristic Nb-L, Nb-Kα, and Nb-Kβ signals belonging to Nb, as well as C-K and O-K peaks, indicates that the MXene structure is embedded in a carbon-based matrix. The low-level Al signal is caused by trace amounts of Al remaining from the MAX phase. The SAED pattern presented in Figure 3e shows the coexistence of bright spot rings and diffuse rings. This pattern confirms that the semi-crystalline nature of the MXene layers and the amorphous character of AC coexist in the MXAC2 composite [45]. The HR-TEM results reveal that MXAC2, which reflects structural integrity, has an advantageous modification in terms of ion transport and electron conduction, playing a critical role in its high electrochemical performance.

**Figure 4:**
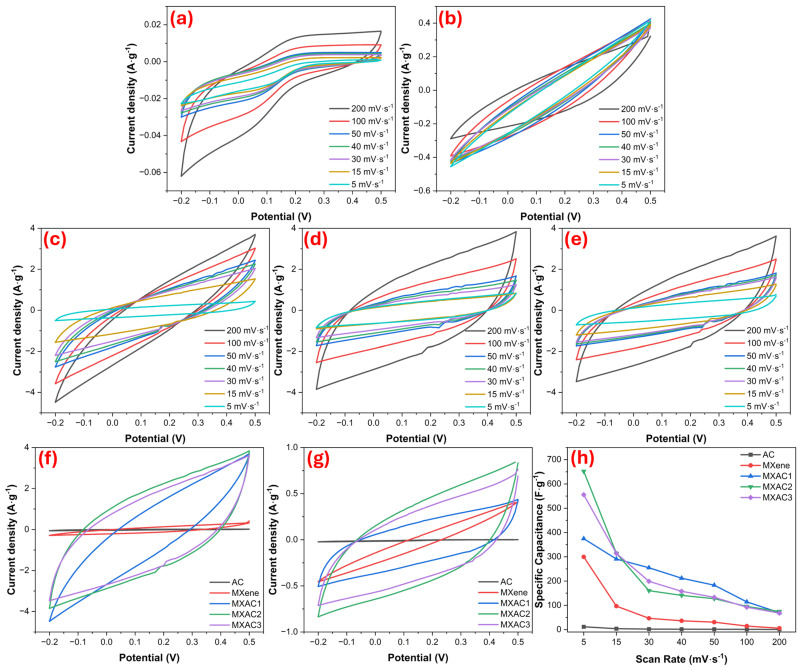
CV curves of (**a**) AC, (**b**) MXene, (**c**) MXAC1, (**d**) MXAC2, and (**e**) MXAC3 at different scan rates; comparative CV profiles at (**f**) 200 mV·s^−1^ and (**g**) 5 mV·s^−1^; and (**h**) specific capacitance as a function of scan rate.

Figure 4 shows a comparative analysis of the CV performance of MXAC1, MXAC2, and MXAC3 electrodes at various scan rates. Figure 4a–e show the CV curves recorded for AC, MXene, MXAC1, MXAC2, and MXAC3, respectively, in the range of 5–200 mV·s^−1^. The CV curves of all samples exhibit a quasi-rectangular shape, indicating that the dominant charge storage mechanism is EDLC in nature [46]. The deviation from the ideal rectangular CV shape is attributed to the combined effect of diffusion-controlled ion transport and pseudocapacitive redox reactions within the porous composite structure. Nb_2_CT_x_ MXene, with its metallic conductivity and surface functional groups (–O, –OH, –F), enables rapid electron transport within the electrode and surface-controlled pseudocapacitive contribution [31]. Furthermore, ion diffusion can also occur because of the restacking tendency arising from the layered structure of Nb_2_CT_x_ MXene [47]. AC’s high specific surface area and microporous structure effectively act as a filler between the MXene layers, significantly reducing this disadvantage [48]. The higher current density observed in the curves, particularly for MXAC2, indicates a larger active surface area and more effective electrode–electrolyte interaction. The MXAC2 electrode has the widest CV area at all scan rates, indicating higher specific capacitance values. The shape of the CV curves is largely preserved as the scan rate increases, indicating that MXAC2 has good ion diffusion and fast charge transfer kinetics [49]. In contrast, in the MXAC1 and MXAC3 samples, the area under the curve increased more limitedly at higher scan rates, and the current response decreased more significantly, especially at high scan rates. It is thought that the performance of MXAC2 is directly related to the 20% AC filling, which increases the distance between the Nb_2_CT_x_ MXene layers and creates shorter and more continuous diffusion pathways for electrolyte ions. For AC, while it primarily provides high capacitive contribution through the EDLC mechanism, the rapid surface redox reactions of Nb_2_CT_x_ MXene support this contribution with pseudocapacitive components [50]. Thus, both high energy storage capacity and high power density can be achieved simultaneously in the MXAC2 composite electrode.

Figure 4f presents comparative CV curves obtained at a scan rate of 200 mV·s^−1^. Even at this high scan rate, the MXAC2 electrode exhibits a higher current response compared to the other samples and better preserves the CV area. This result indicates that MXAC2 maintains its electrochemical stability and conductivity even under high-speed conditions. Furthermore, the increase in active sites on Nb_2_CT_x_ MXene with AC modification contributes particularly to EDLC behavior [51]. Figure 4g shows CV curves obtained at a low scan rate of 5 mV·s^−1^, indicating that MXAC2 exhibits the highest current response even at low speeds and that ions can penetrate the active pores more effectively. Figure 4h presents the specific capacitance values calculated at different scan rates (Table 2). The MXAC2 electrode exhibits the highest specific capacitance, 651.839 F·g^−1^ at 5 mV·s^−1^, and maintains a value of 74.478 F·g^−1^ even at 200 mV·s^−1^, demonstrating superior rate performance. The decreasing specific capacitance values with increasing scan rate are predicted to be due to the internal resistance of the electrodes [52]. In contrast, the specific capacitance values of the MXAC1 and MXAC3 samples were lower and decreased more rapidly with increasing scan rate. These results clearly demonstrate that the synergistic interaction between Nb_2_CT_x_ MXene and AC in the MXAC2 composite significantly improved electrochemical performance by facilitating ion transport [53]. AC can help enhance Faradaic redox reactions on MXAC2 electrode surfaces [54]. As a result, the composite of Nb_2_CT_x_ MXene and AC at 20 wt.%, MXAC2, confirms that these two structural disadvantages are simultaneously eliminated, and the composite electrodes exhibit superior electrochemical performance.

**Figure 5:**
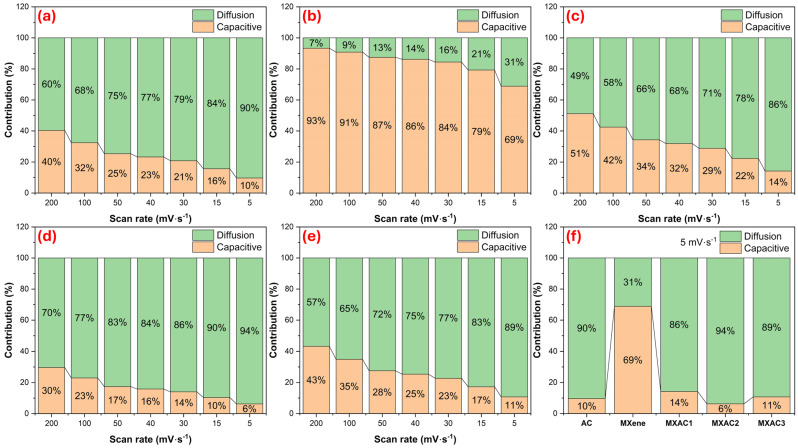
Quantitative calculations of charge storage mechanisms using Dunn’s method for (**a**) AC, (**b**) MXene, (**c**) MXAC1, (**d**) MXAC2, and (**e**) MXAC3 at different scan rates (5–200 mV s^−1^). (**f**) Comparative capacitive and diffusion-controlled contributions of all electrodes at 5 mV s^−1^.

Figure 5 summarizes the separation of capacitive and diffusion-controlled contributions derived from Dunn’s method using CV data collected at different scan rates [55,56]. As seen in Figure 5a, AC is predominantly governed by diffusion-controlled processes, with the diffusion contribution progressively increasing from 60% at 200 mV·s^−1^ to 90% at 5 mV·s^−1^, indicating that charge storage mainly originates from ion transport within the internal porous structure. Conversely, pristine MXene (Figure 5b) exhibits a predominantly capacitive response across the scan rate range studied, with a 93% capacitive contribution at 200 mV·s^−1^ and retaining 69% even at 5 mV·s^−1^. This behavior reflects fast surface-controlled redox reactions and efficient electron transport, consistent with the b values obtained from the power law analysis approaching unity. With the formation of the composites (Figure 5c–e), a gradual transition toward diffusion-dominated kinetics becomes evident. Among them, MXAC2 (Figure 5d) shows the most pronounced diffusion contribution, reaching 94% at 5 mV·s^−1^, which aligns directly with its highest measured specific capacitance. The high diffusion-controlled contribution observed for MXAC2 at low scan rates can be attributed to the intercalation of electrolyte ions into the interlayer galleries of Nb_2_CT_x_. Due to the presence of surface functional groups (–O, –OH, –F), MXene electrodes exhibit pseudocapacitive behavior associated with Faradaic reactions and ion diffusion. In addition, the insertion of AC between the MXene layers partially suppresses restacking and creates more accessible diffusion pathways for electrolyte ions, leading to a diffusion-dominated charge storage process under low-scan-rate conditions. The comparative evaluation at 5 mV·s^−1^ (Figure 5f) further supports this interpretation, where the diffusion contribution follows the order MXAC2 > MXAC3 > MXAC1 > AC > MXene, confirming that the superior electrochemical performance of MXAC2 originates from an optimized hybrid charge storage mechanism combining rapid electron transport with enhanced ion diffusion pathways.

**Figure 6:**
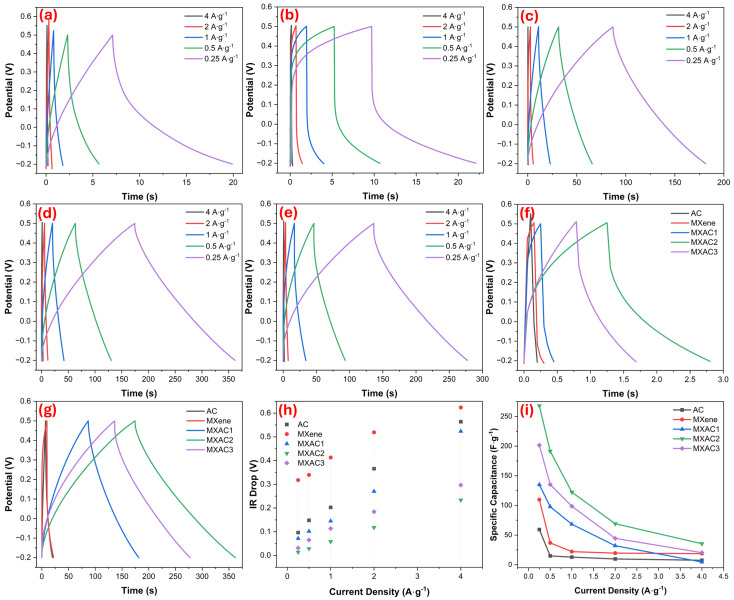
GCD performance: (**a**–**e**) GCD curves recorded for AC, MXene, MXAC1, MXAC2, and MXAC3 electrodes within the range of 0.25–4 A·g^−1^; (**f**) comparative GCD profiles of MXAC electrodes at current densities of 4 A·g^−1^ and (**g**) 0.25 A·g^−1^; (**h**) IR-Drop of electrodes for current densities; (**i**) calculated specific capacitance values at different current densities.

Figure 6a–e show the GCD curves for AC, MXene, MXAC1, MXAC2, and MXAC3, carried out in the range of 0.25–4 A·g^−1^ within the 0.7 V potential window. This demonstrates that, even at relatively high potential windows, electrochemical stability is maintained across all electrodes at high potential ranges at the electrode–electrolyte interface [57]. The highly symmetrical charge and discharge curves for all electrodes indicate that the systems have good electrochemical reversibility and high Coulombic efficiency [58,59]. However, the MXAC2 electrode exhibits higher specific capacitance values, indicating longer discharge times at the same current densities. For example, at 0.25 A·g^−1^, the observed discharge time for MXAC2 is around 350 s, while it is approximately 270 s and 180 s for MXAC3 and MXAC1, respectively. The metallic conductivity of Nb_2_CT_x_ MXene is directly related to the low IR drop values observed in the GCD curves (Figure 6h). This situation indicates that rapid electron transport is occurring within the electrode and internal resistance is being effectively suppressed [60]. In contrast, ion diffusion limitations to restacking, which are only encountered in MXene-based electrodes, have been significantly reduced for AC modifications [61]. The high specific surface area of AC has ensured the preservation of capacitive behavior even at high current densities by creating multiple and short diffusion pathways for electrolyte ions.

In the comparison shown in Figure 6f, conducted at a high current density of 4 A·g^−1^, MXAC2 is seen to offer a significantly longer discharge time compared to MXAC1 and MXAC3. At this current density, the specific capacitance of MXAC2 is maintained at 35.43 F·g^−1^, while for MXAC3 and MXAC1, these values decrease to 20.37 F·g^−1^ and 4.57 F·g^−1^, respectively. Similarly, at a low current density of 0.25 A·g^−1^ in Figure 6g, MXAC2 achieves maximum energy storage performance with a specific capacitance value of approximately 267.93 F·g^−1^; this value is 201.53 F·g^−1^ for MXAC3 and 135.00 F·g^−1^ for MXAC1. While longer discharge times were observed for all samples at 0.25 A·g^−1^, the MXAC2 electrode largely retained its curved form as the current density increased (4 A·g^−1^), indicating that the MXAC2 electrode has superior electrochemical rate performance. This behavior can be explained by the AC’s EDLC contribution, which supports rapid ion adsorption/desorption processes. Aside from that, the surface-controlled pseudocapacitive reactions of Nb_2_CT_x_ MXene remain effective even under high current conditions [62]. Figure 6i presents the calculated specific capacitance values as a function of different current densities, which were plotted from Table 3. The MXAC2 electrode exhibits superior rate performance by maintaining the highest specific capacitance values at all current densities. The capacitance of MXAC2 is maintained at 86.77% when the current density is increased from 0.25 to 4 A·g^−1,^ which is attributed to rapid ion diffusion and low internal resistance. The relatively low capacitance of MXAC1 is attributed to its limited AC filling fraction, while the high AC fraction in MXAC3 is thought to partially suppress the pseudocapacitive contribution of the MXene layers. MXAC2 is considered the most balanced composition for supercapacitor applications, exhibiting high capacitance, good rate performance, and stable electrochemical behavior.

**Figure 7:**
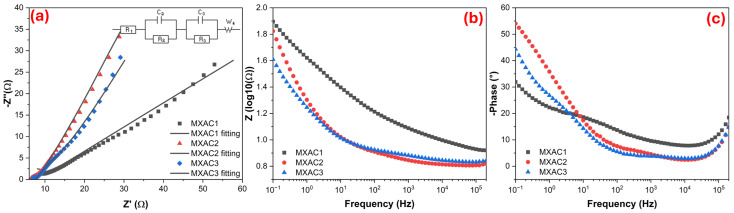
(**a**) Nyquist diagrams, (**b**) impedance-frequency, and (**c**) phase angle–frequency (Bode) plots for MXAC1, MXAC2, and MXAC3 electrodes.

In Figure 7, the EIS results of MXAC1, MXAC2, and MXAC3 electrodes are presented using Nyquist and Bode diagrams, and the obtained data were fitted using the equivalent circuit parameters given in Table 4. It can be seen from the Nyquist diagrams in Figure 7a that all electrodes exhibit a suppressed semicircle in the high-frequency region and a sloping linear character in the low-frequency region. This behavior reveals the simultaneous effect of charge transfer resistance (R_2_:R_ct_) at the electrode/electrolyte interface and the Warburg impedance because of ion diffusion [63,64]. The MXAC2 electrode has a smaller semicircle diameter compared to MXAC1 and MXAC3, which indicates a lower charge transfer resistance. Indeed, the R_2_ value given in Table 4 for MXAC2 was calculated to be only 0.023 Ω; this value was found to be several orders of magnitude lower compared to the MXAC1 (105.03 Ω) and MXAC3 (85.11 Ω) electrodes. This result reveals a significant improvement in electron transfer kinetics at the MXAC2 electrode. This phenomenon is possible because the conductivity of Nb_2_CT_x_ MXene minimizes electron transfer resistance. Furthermore, the porous and hierarchical structure of AC allows for rapid and efficient access of electrolyte ions to active Nb_2_CT_x_ MXene surfaces. Thus, diffusion limitations because of restacking, frequently observed in MXene layers, are significantly suppressed by the addition of AC [65]. Comparing the series resistance (R_1_) values, a value of 6.744 Ω was obtained for MXAC2, which exhibits a similar but lower internal resistance profile compared to MXAC1 (7.364 Ω) and MXAC3 (6.892 Ω). This situation indicates that in MXAC2, the MXene-AC interface improves the quality of electrical contact [66]. Additionally, the capacitive elements C_2_ and C_3_ determined for MXAC2 are 86.35 × 10^−3^ F and 7276 × 10^−3^ F, respectively, indicating a higher capacitive contribution compared to MXAC1 and MXAC3.

In the Bode |Z|-frequency plot presented in Figure 7b, the MXAC2 electrode exhibits the lowest impedance values in the medium- and high-frequency regions, demonstrating a faster frequency response and low internal resistance characteristics. Similarly, in the phase angle–frequency curve in Figure 7c, MXAC2 exhibited more pronounced capacitive behavior compared to MXAC1 (31.90°) and MXAC3 (44.34%), with the phase angle reaching 54.02° at low frequencies. This situation indicates that the dominant EDLC mechanism of AC is effectively supported by the surface-controlled pseudocapacitive contributions of Nb_2_CT_x_ MXene [67]. To further investigate the ion transport kinetics within the electrode materials, the ion diffusion coefficients (D) were quantified using the Randles plot analysis in the low-frequency Warburg region. The D values were calculated as 3.37 × 10^−13^ cm^2^·s^−1^ for MXAC1, 1.04 × 10^−12^ cm^2^·s^−1^ for MXAC2, and 6.78 × 10^−13^ cm^2^·s^−1^ for MXAC3. The notably higher diffusion coefficient of MXAC2 indicates that the optimization facilitates a lower resistance to ion migration and more efficient electrolyte penetration into the porous structure. This quantitative finding is in excellent agreement with the lower charge transfer resistance and superior rate capability observed for the MXAC2 electrode. The balanced Nb_2_CT_x_ MXene/AC fraction of MXAC2 demonstrates its optimized ion diffusion, low charge transfer resistance, and enhanced capacitive properties, making it the most suitable electrode for supercapacitor applications.

**Figure 8:**
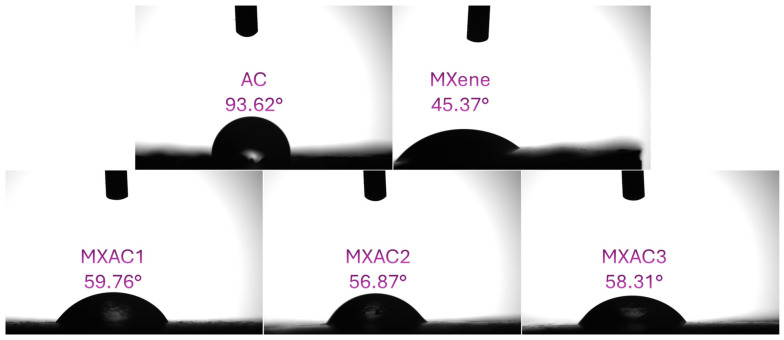
Static water contact angle images of AC, MXene, and MXAC composite electrodes.

Figure 8 shows the static water contact angle measurements of AC, Nb_2_CT_x_ MXene, and MXAC composites. Pristine AC exhibits a relatively high contact angle (93.62°), indicating limited wettability, which can restrict electrolyte penetration and reduce effective electrochemical utilization. In contrast, Nb_2_CT_x_ displays a much lower contact angle (45.37°) due to its hydrophilic surface terminations (–O, –OH, –F), facilitating rapid ion transport and improved capacitive behavior. The MXAC composites present intermediate contact angles (59.76° for MXAC1, 56.87° for MXAC2, and 58.31° for MXAC3), confirming enhanced wettability compared to pure AC. Among them, MXAC2 exhibits the lowest contact angle, correlating with its superior electrochemical performance. Improved wettability enhances electrolyte accessibility, reduces interfacial resistance, and promotes efficient ion diffusion within the porous framework. These findings are consistent with the electrochemical results and Dunn’s method analysis, highlighting the critical role of optimized surface wettability in enhancing charge storage performance.

**Figure 9:**
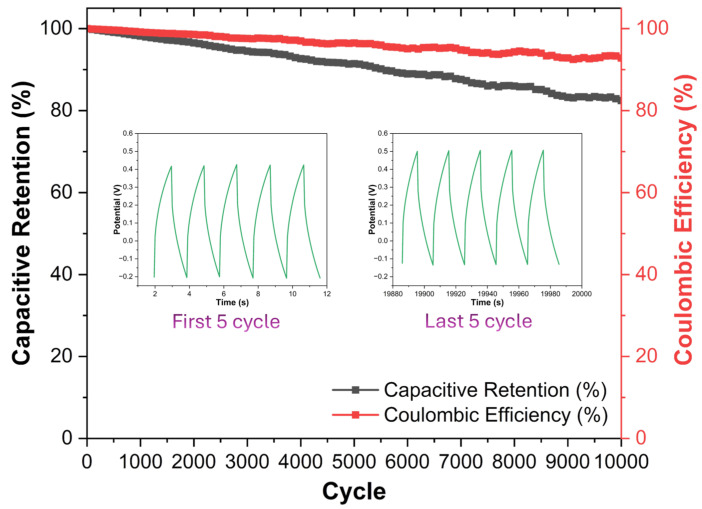
Capacitive retention and Coulombic efficiency behavior of MXAC electrodes over 10,000 cycles with GCD profiles for the initial/final 5 cycles as an inset.

Figure 9 shows the capacitive resistance and coulombic efficiency behavior of the MXAC2 electrode during long-term GCD tests. The MXAC2 electrode exhibited quite high cycle stability, demonstrating 82.15% capacity retention after 10,000 cycles. The coulombic efficiency remains constant at 92.45% throughout all cycles, indicating that the charging and discharging processes occur with high reversibility and that there is no significant degradation in the electrode structure [68]. The surface functional groups of Nb_2_CT_x_ MXene form a strong interface with the electrolyte, ensuring the sustainability of surface-controlled pseudocapacitive reactions over cycles [69,70]. However, in pure MXene systems, capacitance loss can be observed during cycling to restacking and structural relaxation. The porous carbon skeleton of AC, which has high mechanical stability, physically supports the MXene layers, suppressing structural degradation and maintaining electrode integrity for a long time [71]. The more stable trend exhibited by MXAC2 could be a result of its direct compatibility with the low charge transfer resistance (R_2_ = 0.023 Ω) determined in EIS analyses. GCD curves for the first and last 5 cycles given in the in-figure insets clearly confirm that structural and electrochemical stability is maintained in the MXAC2 electrode throughout the cycles. Therefore, the combined use of Nb_2_CT_x_ MXene and AC demonstrates a significant synergistic effect not only in terms of high initial performance but also in terms of long-term electrochemical stability.

As clearly evidenced in Table 5, the MXAC2 composite exhibits excellent electrochemical performance among recently reported Nb_2_CT_x_-based and biomass-derived carbon electrodes. In particular, MXAC2 delivers a high specific capacitance of 267.9 F·g^−1^ in 1 M KOH, which is comparable to or higher than several previously reported Nb_2_C-based hybrid systems, such as biomass-derived Nb_2_CT_x_/AC composites (285.6 F·g^−1^) [21] and palm-flower-derived AC hybrids (296.0 F·g^−1^) [59]. The competitive capacitance performance indicates an efficient electrochemical utilization of the available surface area and effective charge transfer within the MXene–carbon framework. In addition, the MXAC2 electrode demonstrates a capacity retention of 92.4% after 10,000 cycles, confirming its excellent long-term electrochemical stability. The improved electrochemical performance can be attributed to the synergistic interaction between Nb_2_CT_x_ nanosheets and the porous biomass-derived activated carbon, which effectively mitigates MXene restacking while providing abundant ion-accessible pathways and electrochemically active sites.

**Figure 10:**
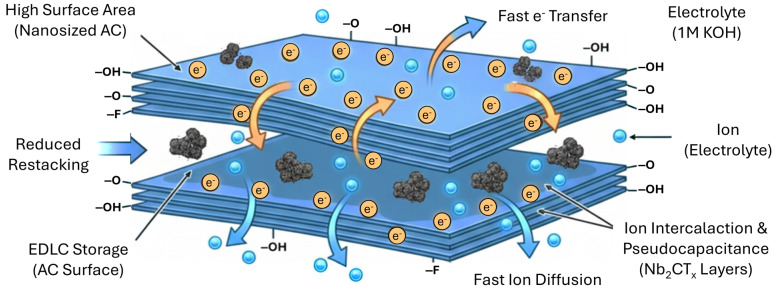
Charge–discharge mechanism for nano-sized AC modified Nb_2_CT_x_ MXene electrodes.

The dominant EDLC contribution of AC supports rapid charge–discharge capability at high power densities, while the pseudocapacitive character of Nb_2_CT_x_ MXene may result in increased energy density, as illustrated in Figure 10 [51]. Both long cycle life and high energy-power performance are achieved simultaneously through a balanced microstructure established between MXene and AC in the MXAC2 electrode. Specifically, the low internal resistance, high coulombic efficiency, and stable GCD profiles clearly demonstrate that MXAC2 is the most promising electrode for practical supercapacitor applications.

## 4. Conclusions

Structural and electrochemical analyses have shown that the addition of AC suppresses the restacking of MXene layers and creates a favorable hierarchical porous structure for ion diffusion. Specifically, the MXAC2 composite stood out as the sample that achieved the most balanced synergy between MXene and AC. Nyquist diagrams obtained from EIS analysis and equivalent circuit modeling revealed that the MXAC2 electrode has a very low charge transfer resistance (0.023 Ω). This value indicates a significant improvement in ion and electron transport kinetics compared to the MXAC1 (107 Ω) and MXAC3 (85.11 Ω) samples. Additionally, the higher capacitance value for MXAC2 (1316 × 10^−3^ F) indicates improved charge storage capacity at the electrode–electrolyte interface. The higher phase angle of MXAC2 in the low-frequency region of Bode diagrams indicates dominant capacitive behavior and rapid ion diffusion. In long-term cycle stability tests, the MXAC2 electrode demonstrated high electrochemical stability by exhibiting 82.15% capacity retention and 92.45% coulombic efficiency after 10,000 cycles. The almost overlapping GCD curves obtained in the first and last 5 cycles indicate that structural degradation is negligible. In conclusion, the MXAC2 electrode, with its low internal resistance and excellent cycling stability, clearly demonstrates its strong potential as a sustainable and high-performance supercapacitor application for biodegradable waste-based MXene/AC composites.

## Figures and Tables

**Table 1:** Elemental composition of banana peel-derived activated carbon (AC) determined by CHNS analysis.

	C	H	N	S	O
Amount (%)	64.93	2.97	3.13	1.76	27.21

**Table 2:** Specific capacitances from CV at various scan rates on the AC, MXene, MXAC1, MXAC2, and MXAC3 electrodes in 1.0 M KOH electrolyte at 0.7 V.

Scan Rate (mV·s^−1^)	AC (F·g^−1^)	MXene (F·g^−1^)	MXAC1 (F·g^−1^)	MXAC2 (F·g^−1^)	MXAC3 (F·g^−1^)
5	11.429	299.547	374.484	651.839	555.500
15	3.743	96.621	290.555	314.151	313.687
30	2.038	46.591	254.631	160.372	198.836
40	1.583	36.199	211.827	140.890	157.648
50	1.359	30.565	182.835	127.507	133.089
100	0.965	14.540	114.001	95.284	92.213
200	0.695	5.719	69.854	74.478	67.941

**Table 3:** Specific capacitances from GCD curves at different current densities on the AC, MXene, MXAC1, MXAC2, and MXAC3 electrodes in 1.0 M KOH electrolyte.

Current Density (A·g^−1^)	AC (F·g^−1^)	MXene (F·g^−1^)	MXAC1 (F·g^−1^)	MXAC2 (F·g^−1^)	MXAC3 (F·g^−1^)
0.25	59.32	109.61	135.00	267.93	201.53
0.5	14.90	36.81	97.86	191.43	135.20
1	12.80	22.10	68.29	122.29	98.57
2	9.74	19.53	32.00	69.14	44.47
4	7.49	18.69	4.57	35.43	20.37

**Table 4:** Specific capacitances from GCD curves at different current densities on the MXAC1, MXAC2, and MXAC3 electrodes in 1.0 M KOH electrolyte.

Electrodes	R_1_ (Ω)	R_2_ (Ω)	C_2_ (10^−3^, F)	R_3_ (Ω)	C_3_ (10^−3^, F)	W_4_ (Ω·s^−1/2^)
MXAC1	7.364	105.03	15.50	1.241	324	57.18
MXAC2	6.744	0.023	86.35	1316	7276	97.93
MXAC3	6.892	85.11	32.31	0.721	715	75.96

**Table 5:** Comparison of electrochemical performance metrics (specific capacitance and capacity retention) of the MXAC2 composite with other Nb_2_CT_x_-based and biomass-derived activated carbon electrode materials reported in the literature.

Electrode Material	Electrolyte	Specific Capacitance (F·g^−1^)	Capacity Retention (%)	Ref.
Biomass-derived AC (N-doped)	1 M KCl	98.0 at 0.2 A·g^−1^	92.5 (1000 cycles) at 1.6 A·g^−1^	[14]
Ti_3_C_2_T_x_/AC Flexible Composite	H_2_SO_4_/PVA	99.7 at 10 A·g^−1^	92.4 (10,000 cycles) at 10 A·g^−1^	[20]
Nb_2_CT_x_/Biomass-derived AC	1 M H_2_SO_4_	285.6 at 0.5 A·g^−1^	92.9 (5000 cycles) at 10 A·g^−1^	[21]
Palm Flower-Derived AC	1 M H_2_SO_4_	296.0 at 0.5 A·g^−1^	90.0 (2000 cycles) at 8 A·g^−1^	[59]
MXAC2	1 M KOH	267.9 at 0.25 A·g^−1^	92.4 (10,000 cycles) at 4 A·g^−1^	This Work

## Data Availability

The data presented in this study are available on request from the corresponding author.
